# Cross-sectional chest circumference and shape development in infants

**DOI:** 10.1186/s13104-022-06087-z

**Published:** 2022-06-15

**Authors:** Nima Seifnaraghi, Serena de Gelidi, Inéz Frerichs, Merja Kallio, Erich Sorantin, Andrew Tizzard, Andreas Demosthenous, Richard H. Bayford

**Affiliations:** 1 Department of Natural Sciences, Middlesex University, London, UK; 2 Department of Anaesthesiology and Intensive Care Medicine, University Medical Centre Schleswig-Holstein, Kiel, Germany; 3 PEDEGO Research Unit, Medical Research Center, Department of Children and Adolescents, Oulu University Hospital, Oulu, Finland; 4 Department of Radiology, Medical University of Graz, Graz, Austria; 5 Department of Electronic and Electrical Engineering, University College London, London, UK

**Keywords:** Chest circumference, Neonatal chest development, Cross-sectional chest shape

## Abstract

**Objective:**

This study investigates the development of the thoracic cross-section at the nipple line level during the early stages of life. Unlike the descriptive awareness regarding chest development course, there exist no quantitative references concerning shape, circumference and possible dependencies to age, gender or body weight. The proposed mathematical relations are expected to help create guidelines for more realistic modelling and potential detection of abnormalities. One potential application is lung electrical impedance tomography (EIT) monitoring where accurate chest models are crucial in both extracting reliable parameters for regional ventilation function and design of EIT belts. Despite their importance, such reference data is not readily available for the younger age range due to insufficient data amid the regulations of neonatal imaging.

**Results:**

Chest circumference shows the highest correlation to body weight following the relation $f\left(x\right)= 18.3735 \ \mathrm{ln}\left(0.0012x+2.1010\right)$ where *x* is the body weight in grams and *f(x)* is the chest circumference in cm at the nipple line level. No statistically significant difference in chest circumference between genders was detected. However, the shape indicated signs of both age and gender dependencies with on average boys developing a more rectangular shape than girls from the age of 1 years and 9 months.

**Supplementary Information:**

The online version contains supplementary material available at 10.1186/s13104-022-06087-z.

## Introduction

Information about chest development in infants regarding circumference and shape are quite rare [1]. While clinicians can empirically observe that newborn babies have a more circular shape than the more rectangular shape seen in later years, there is no quantitative reference to support this observation. Unlike anthropometric parameters such as head circumference used as an indicator of normal growth, no direct clinical relation is reported about chest circumference and shape in relation to age, gender, body height or weight.

One of the fields which would benefit from the information presented in this work is chest electrical impedance tomography (EIT). EIT is a non-invasive imaging modality for monitoring air volume changes in lungs during breathing.

The images are reconstructed based on measured electric potentials from an EIT belt wrapped around a patient’s chest. The initial assumed model for chest boundary at the level of belt placement affects the accuracy of generated images [2]. Ideally the image reconstruction algorithm should use realistic patient specific models. This type of model preparation is obtainable using a combination of sensors like gravitation sensors [3] when measured superficially or via magnetic resonance imaging (MRI) or computed tomography (CT) scans. The required equipment to conduct the external detection is not often available and the latter cases become rare within the considered age group amid the vulnerability of infants to high radiation dosage in CT-scans [4] or necessity of sedation during MRI. Therefore, to obtain a realistic estimation of chest contour extra information is needed.

The quantitative comparison of geometries when they do not follow a regular pattern is not a straight forward procedure [5]. In this work, a value proportional to the ratio of ventro-dorsal distance to lateral chest width was defined as an indicator for shape. The values closer to 1 refer to more circular shapes whereas lower values correspond to a flatter rectangular shape. It is of note that in morphological studies often shape is independent of any size (scale) and instead the domain containing both shape and size is recognized as form domain. Analyzing shape rather than form minimizes the possible distortion of parameters such as genetic and nutritional differences to reach a more meaningful conclusion. Each of these features was tested against weight, gender and age to discover the contributing variable(s).

## Main text

### Method

In this study two independent datasets were used for chest circumference and shape investigations. The first dataset, consisting of 201 patients, was obtained by trained nurses measuring with tape measures at the under arms level. Among these patients 43 cases were recorded at the Archbishop Makarios III Hospital, Nicosia, Cyprus (Ethics number: EEBK/EP/2016/32), 88 at the Emma Children’s Hospital, Amsterdam, Netherlands (Ethics number: METC 2016/184), and 70 at the Oulu University Hospital, Oulu, Finland (Ethics number: EETTMK 35/2017).

The data were fitted with a logarithmic curve using the least squared error method providing a relationship between weight in grams and chest circumference in cm.

The curve was firstly fitted to the whole population, corresponding to an age range from post-menstrual age of 27 weeks at 700 g to 2.7 years at 10 kg. To evaluate any gender dependency, two extra gender specific curves were calculated. The fitted curves were verified for their capability of explaining the data using the best fit technique.

The data for studying the thorax cross-sectional shape was obtained via transversally slicing the 3D models created from CT-scan images of 62 patients in the range of 0–7 years retrieved from the image archive at the Medical University of Graz with permission of the university’s ethics board (28-540 ex 15/16).

One factor causing undesired variation in the cross-sectional contour is the position of the arms relative to the trunk resulting from the scapula bone movement during arm elevation, known as scapula rhythm [6] adding lateral bulges at the dorsal region by rotating outwards. Seven patients were removed as they did not meet this criterion with their arms placed beside their bodies. Leaving 33 male and 22 female patients. The details of this set are provided in Additional file 1: Table S1.

Another factor to be considered before transversally slicing the 3D models is the relative position of the patient to the spiral travelling pattern of the source/detector along axial direction during the scan. Despite the restricted space of CT-scan gantry and being limited to supine position, still three potential rotations are possible, occurring either individually or combined. These variations are typically caused by additional supporting materials (pillow, mattress, etc.) during the placement of the patient on the monitoring platform.

In order to compensate for the position-related artefacts, anatomical landmarks were exploited. Nipples were selected as the anterior landmarks whereas the 6th paravertebral intercostal space was chosen on the posterior side. Considering the nipple movement on the surface of the thorax with elevation of arms, the anterior landmarks were always checked against the symmetry of the ribs on the transversal plane at the sternum side and adjusted accordingly. To constrain the irregular shape of the chest contour at the desired level, proceeding the creation of consistent 2D cross-sections, each model was fitted with a minimum surface area trapezoid capable of enclosing the model. This type of polygon was preferred to trivial rectangular bounding boxes as they reveal more information regarding the shape, and more importantly, are less sensitive to insignificant geometrical imperfections.

The ratio of height to base of each trapezoid was calculated and considered against the corresponding patient’s age. The higher values of this ratio indicate a more circular cross-section and conversely the lower values represent more flattened rectangular shapes.

To form the desired trapezoids, discrete sample points were taken from each cross-sectional contour using a small Euclidean distance to enable accurate tracking of the continuous geometry shape.

The yielded sample points were classified to pixels of a grid. When more than one point was assigned to a certain pixel they were replaced by their mean coordinates as the representative of that pixel.

**Fig. 1:**
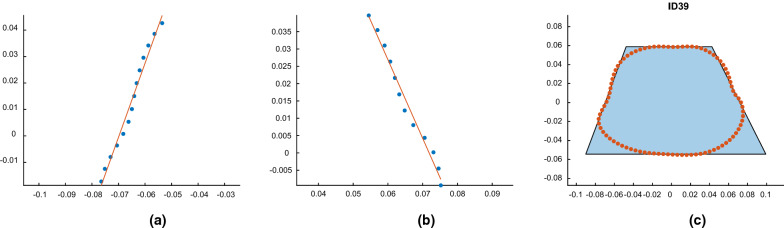
**a** Line fitted to patients right lateral points, **b** line fitted to patients left lateral points, **c** fitted trapezoid

The top and bottom rows were identified as the anterior and posterior sides respectively. In order to detect the pixels corresponding to lateral sides, a selection criterion was introduced to allow the selection of pixels falling within certain bounds. These bounds were defined as the row with the widest lateral distance at the posterior side and the row consisting of the corner points at the anterior side. The corner points were allocated to the pixels where the gradient of the path traveling from one pixel to the closest ventrally located neighboring pixel exceeded a threshold of 45 degrees. These gradients indicated the termination of the path on lateral sides and approach to the flatter anterior side. The lateral presenting datasets were then fitted with a linear line using the least squared error method [7]. An example of the lateral fitted lines for a thoracic cross-section of a male patient at 9 months of age are shown in Fig. 1a and b for the right and left side of the patient respectively where Fig. 1c demonstrates the final enclosing trapezoid.

## Results

### A. Chest circumference

The function explaining chest circumference to the body weight was evaluated to be

1 $$f\left(x\right)= 18.3735 \ \mathrm{ln}\left(0.0012x+2.1010\right).$$

**Fig. 2:**
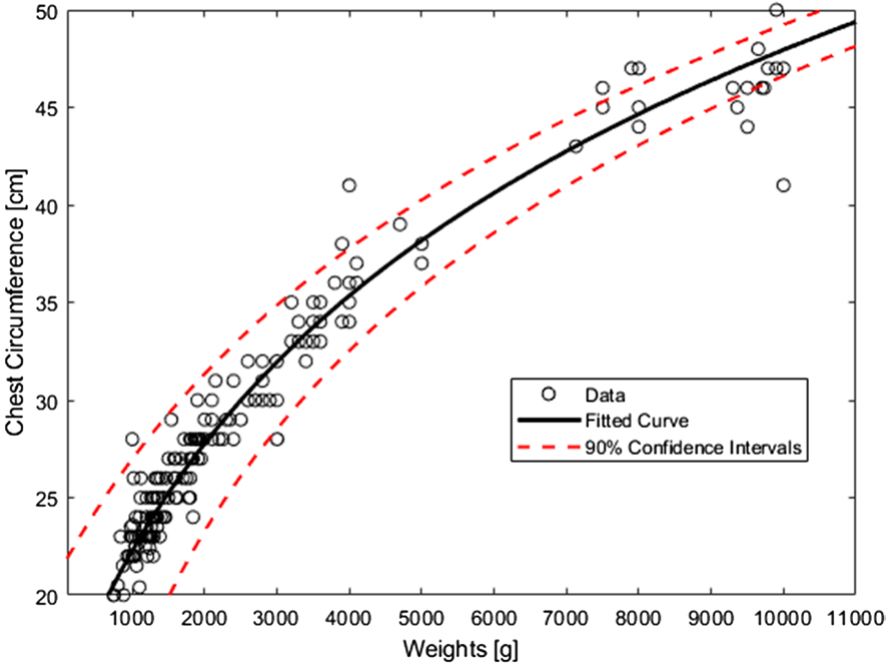
Logarithmic curve relating chest circumference in cm to weight in g with R^2^ = 0.96 obtained in the whole population of 201 patients

With variable *x* representing body weight in grams and $f\left(x\right)$ the estimated chest circumference in cm. The computed corresponding confidence intervals of 90% for new observations are plotted in Fig. 2. The best fit was evaluated to be 0.96. To examine the role of gender, two additional curves were developed by fitting separately to the boys’ and girls’ population data.

However, the maximum difference between these gender specific plots was less than 2 cm which only occurred at the very beginning of the considered interval.

### B. Shape development

Whereas the chest circumference did not show any significant dependencies to age or gender, the shape of chest cross-section seemed to be a function of both. The computed ratios from the 55 CT-scan case studies as an indicator of shape change were fitted with a 1st degree rational function of the form:

2 $$y= \frac{{c}_{1}x+{c}_{2}}{x+{c}_{3}}$$

where *x* is the age in years and $y$ is the dimensionless estimated ratios. For female cases the coefficients were calculated to be 0.41096, 2.1641and 3.5342 for ${c}_{1}$, ${c}_{2}$ and ${c}_{3}$ respectively while the same coefficients changed to 0.12662, 5.7865 and 9.2355 when the male population was considered.

**Fig. 3:**
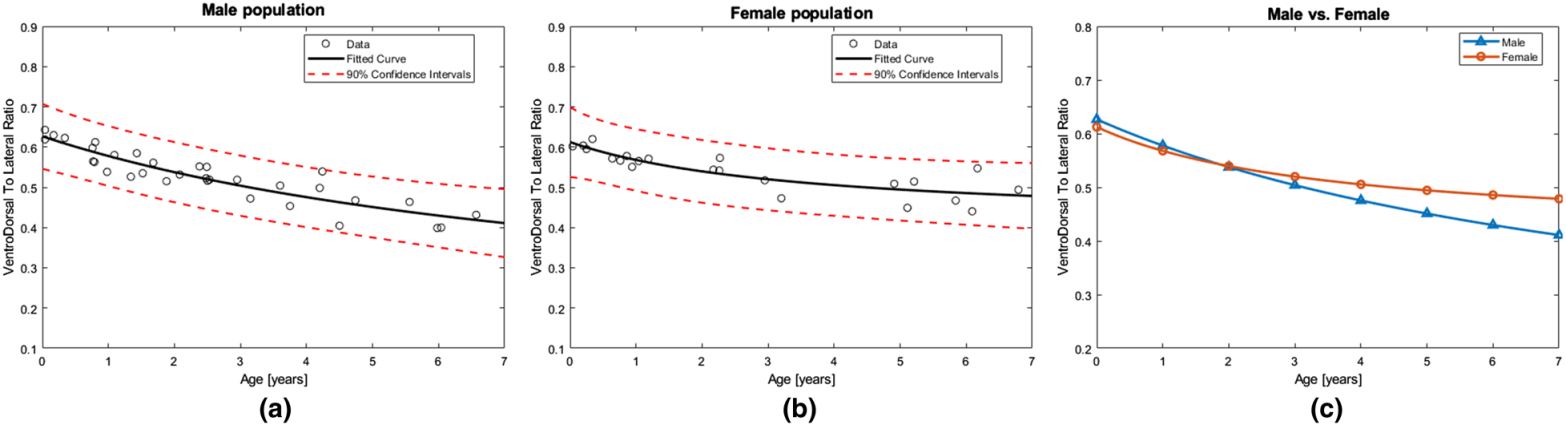
Logarithmic curve relating ventro-dorsal to lateral ratio in fitted trapezoids **a** boys R^2^ = 0.834, **b** girls R^2^ = 0.745, **c** superimposed curves

Figure 3a and b plot the ratio values along with the fitted curve for each gender as a function of age with their 90% predicted confidence intervals. Figure 3c shows the superimposition of these curves to highlight the different paths during growth according to their gender. It is evident from the plot that both genders start their trend with a roundish shape at above 60% and continue toward a rectangular shape similarly until nearly 2 years of age. However, from this point onwards boys accelerate to a more rectangular shape while the girls maintain a slower pace. The intersection of the plots at 1.9 years is consistent with earlier findings [8] regarding the start of taking the adult shape on mid-thoracic level near the end of the second postnatal year.

## Discussion

The data suggests the chest circumference can be well explained with a logarithmic expression as a function of weight. However, gender does not seem to have an impact on the considered age range since the gender specific logarithmic curves matched closely and had their highest difference at weights below 1000 g where data points were not sufficiently dense. In addition, the chest circumference data was compared against age, but no apparent relation was observed.

Thus, it can be asserted weight is playing more significant role compared to age and gender at this age group.

During these experiments the choice of trapezoid shape proved to be superior to rectangular bounding boxes as any imperfections caused naturally or by asymmetric arm elevation levels could directly affect the fitted box while using the whole lateral points was less sensitive to insignificant displacements.

## Conclusion and outlook

By discovering the anatomical relations in neonates and small children more specific devices could be designed for this age range. For instance, while EIT is being frequently used in the neonatal and pediatric intensive care units and delivery rooms [9, 10], still the image reconstruction in neonates and infants is impeded by the lack of appropriate chest and lung models compared to adults. An example of EIT image reconstruction based on simulated EIT signal from a 2.5-month old male weighing 4970 g is presented in Additional file 2: Fig. S1. A realistic model was generated according to the proposed method (Additional file 2: Fig. S1.c) in comparison to the scaled down model of an older patient at 6.57 years representing the typical approach. The designed trapezoid model had 0.63 height to base ratio and chest circumference of 38.35 cm calculated from Eq. (1) whereas the true value from the CT scan was 39.51 cm. The fitted trapezoid was further rounded with a radius corresponding to 25% of its height at all corners. The impact of using inaccurate existing models on clinically important parameters in lung monitoring is detailed in [11].

## Limitations

Despite the chest circumference that is based on 201 patients, the shape changes were derived from merely 55 cases due to the rarity of CT-scans at this age range. The effect is reflected in wider prediction confidence intervals and lower best fit, especially in female cases where they were only 22 samples. Therefore, this study should be updated with more samples as new data becomes available. Nonetheless, the present findings can be regarded as a primary guide for further investigations of cross-sectional thoracic shape changes in childhood.

## Supplementary Information

**Additional file 1: Table S1.** The gender, age, weight and chest circumference at under arm level for the 55 patients used to develop the parameters necessary for creating an age appropriate 2D chest models.

**Additional file 2: Fig. S1.** Example of EIT image reconstruction, (a) superimposed lungs from the 3D model, (b) scaled down 2D model of a 6.57 years old, (c) trapezoid model generated.

## Data Availability

Unfortunately, the datasets analysed during the current study are not publicly available due to the limitation of the projects regarding publicity of the raw clinical data. The model designs were generated using Solidworks (3DS Dassault Systèmes, Vélizy-Villacoublay, France) and Comsol multiphysics software (COMSOL AB, Stockholm, Sweden). The analytical parts were done using Matlab software (MathWorks, Natick, USA).
