# Supplementary material for: Cross-sectional chest circumference and shape development in infants
Source: BMC Res Notes. 2022 Jun 15;15:206. doi: 10.1186/s13104-022-06087-z (PMC9202117; doi:10.1186/s13104-022-06087-z)
Supplement: Supplementary file 1 — Additional file 1: Table S1. The gender, age, weight and chest circumference at under arm level for the 55 patients used to develop the parameters necessary for creating an age appropriate 2D chest models. [file 13104_2022_6087_MOESM1_ESM.docx]

**Table S1 The gender, age, weight and chest circumference at under arm level for the 55 patients used to develop the parameters necessary for creating an age appropriate 2D chest models.**

| **ID** | **Gender**  **(m/f)** | **Age Range**  **(years)** | **Weight**  **(grams)** | **Chest**  **Circumference (m)** |
| --- | --- | --- | --- | --- |
| 1 | m | 0-0.5 | 4970 | 0.3951 |
| 2 | f | 6-6.5 | 22000 | 0.5886 |
| 3 | m | 4.5-5 | 16000 | 0.5875 |
| 4 | m | 0.5-1 | 10000 | 0.4735 |
| 5 | m | 5-5.5 | 21000 | 0.6415 |
| 6 | m | 1-1.5 | 8080 | 0.4476 |
| 7 | m | 1-1.5 | 10200 | 0.4710 |
| 8 | f | 0.5-1 | 9360 | 0.4672 |
| 9 | f | 0.5-1 | 9300 | 0.4636 |
| 10 | f | 0.5-1 | 10000 | 0.4064 |
| 11 | f | 3-3.5 | 14500 | 0.5704 |
| 12 | f | 4.5-5 | 14500 | 0.5443 |
| 13 | m | 2.5-3 | 16400 | 0.5307 |
| 14 | f | 0-0.5 | 1404 | 0.2610 |
| 15 | m | 1.5-2 | 12000 | 0.5192 |
| 16 | w | 5-5.5 | 20000 | 0.6039 |
| 17 | m | 2-2.5 | 11000 | 0.5025 |
| 18 | m | 1.5-2 | 8000 | 0.4610 |
| 19 | f | 2.5-3 | 16600 | 0.5310 |
| 20 | m | 2-2.5 | 12000 | 0.4937 |
| 21 | f | 5.5-6 | 17500 | 0.5746 |
| 22 | m | 0.5-1 | 8000 | 0.4300 |
| 23 | m | 0-0.5 | 7130 | 0.4339 |
| 24 | f | 6-6.5 | 18500 | 0.5702 |
| 25 | m | 5.5-6 | 20000 | 0.5578 |
| 26 | f | 5-5.5 | 17600 | 0.5538 |
| 27 | f | 2-2.5 | 10000 | 0.4709 |
| 28 | m | 0-0.5 | 2711 | 0.3034 |
| 29 | m | 1-1.5 | 10400 | 0.4809 |
| 30 | f | 1-1.5 | 9785 | 0.4657 |
| 31 | w | 1-1.5 | 7900 | 0.4643 |
| 32 | w | 0-0.5 | 3245 | 0.3491 |
| 33 | m | 0.5-1 | 9650 | 0.4874 |
| 34 | f | 0-0.5 | 5880 | 0.4045 |
| 35 | m | 0-0.5 | 4316 | 0.3888 |
| 36 | m | 2-2.5 | 14000 | 0.5167 |
| 37 | w | 6.5-7 | 20200 | 0.5914 |
| 38 | f | 2-2.5 | 13800 | 0.5181 |
| 39 | m | 4-4.5 | 13700 | 0.5559 |
| 40 | m | 2-2.5 | 11000 | 0.4818 |
| 41 | m | 6-6.5 | 24000 | 0.6493 |
| 42 | m | 6.5-7 | 20600 | 0.5936 |
| 43 | f | 0-0.5 | missing | 0.3627 |
| 44 | m | 0.5-1 | missing | 0.4873 |
| 45 | f | 0.5-1 | missing | 0.4543 |
| 46 | f | 2-2.5 | missing | 0.5125 |
| 47 | m | 0.5-1 | missing | 0.4943 |
| 48 | m | 2.5-3 | missing | 0.5087 |
| 49 | m | 1.5-2 | missing | 0.5499 |
| 50 | m | 3.5-4 | missing | 0.5508 |
| 51 | m | 4.5-5 | missing | 0.5827 |
| 52 | m | 4-4.5 | missing | 0.6247 |
| 53 | m | 2.5-3 | missing | 0.5052 |
| 54 | m | 3.5-4 | missing | 0.5981 |
| 55 | m | 3-3.5 | missing | 0.5151 |
