# Supplementary material for: Cross-sectional chest circumference and shape development in infants
Source: BMC Res Notes. 2022 Jun 15;15:206. doi: 10.1186/s13104-022-06087-z (PMC9202117; doi:10.1186/s13104-022-06087-z)
Supplement: Supplementary file 2 — Additional file 2: Fig. S1. Example of EIT image reconstruction, (a) superimposed lungs from the 3D model, (b) scaled down 2D model of a 6.57 years old, (c) trapezoid model generated. [file 13104_2022_6087_MOESM2_ESM.docx]

| 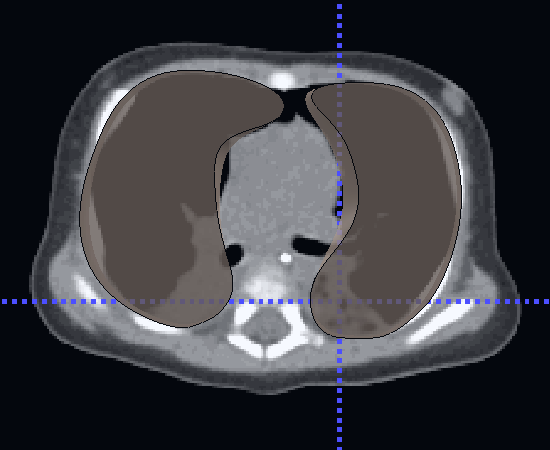  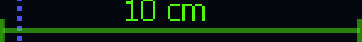 | 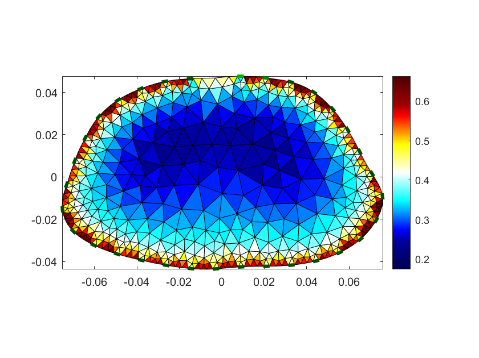 | 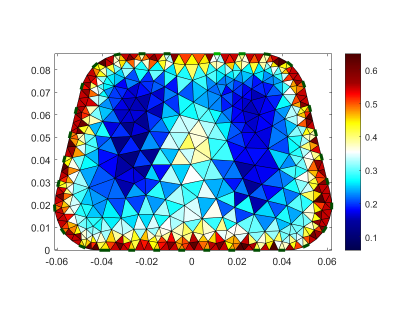 |
| --- | --- | --- |
| (a) | (b) | (c) |
| **Fig. S1** Example of EIT image reconstruction, (a) superimposed lungs from the 3D model, (b) scaled down 2D model of a 6.57 years old, (c) trapezoid model generated. | | |
